# Clinical and metabolic response to vitamin D plus probiotic in schizophrenia patients

**DOI:** 10.1186/s12888-019-2059-x

**Published:** 2019-02-21

**Authors:** Amir Ghaderi, Hamid Reza Banafshe, Naghmeh Mirhosseini, Mohamad Moradi, Mohammad-Amin Karimi, Fateme Mehrzad, Fereshteh Bahmani, Zatollah Asemi

**Affiliations:** 1 Department of Addiction Studies, School of Medical, Kashan University of Medical Sciences, Kashan, I.R Iran; 2 Department of Pharmacology, School of Medicine, Kashan University of Medical Sciences, Kashan, I.R Iran; 3 Physiology Research Center, Kashan University of Medical Sciences, Kashan, I.R Iran; 4 School of Public Health, University of Saskatchewan, Saskatoon, SK Canada; 5 Department of Psychiatry School of Medicine, Kurdistan University of Medical Science, Kurdistan, Iran; 6 Department of Educational Sciences, Science and Research Branch, Islamic Azad University, Tehran, Iran; 7 Department of Psychiatry, School of Medicine, Kashan University of Medical Science, Kashan, I.R Iran; 8 Research Center for Biochemistry and Nutrition in Metabolic Diseases, Kashan University of Medical Sciences, Kashan, I.R Iran

**Keywords:** Vitamin D, Probiotic supplementation, Metabolic status, Schizophrenia

## Abstract

**Background:**

This study determined the effects of a novel combination of vitamin D and probiotic on metabolic and clinical symptoms in chronic schizophrenia.

**Methods:**

This trial was conducted among 60 patients with chronic schizophrenia to receive either 50,000 IU vitamin D3 every 2 weeks plus 8 × 10^9^ CFU/day probiotic (*n* = 30) or placebo (n = 30) for 12 weeks.

**Results:**

Vitamin D and probiotic co-supplementation was associated with a significant improvement in the general (− 3.1 ± 4.7 vs. + 0.3 ± 3.9, *P* = 0.004) and total PANSS scores (− 7.4 ± 8.7 vs. -1.9 ± 7.5, *P* = 0.01). Vitamin D and probiotic co-supplementation also significantly increased total antioxidant capacity (+ 51.1 ± 129.7 vs. -20.7 ± 53.3 mmol/L, *P* = 0.007), and significantly decreased malondialdehyde (− 0.3 ± 0.9 vs. + 0.2 ± 0.4 μmol/L, *P* = 0.01) and high sensitivity C-reactive protein levels (− 2.3 ± 3.0 vs. -0.3 ± 0.8 mg/L, *P* = 0.001) compared with the placebo. Moreover, taking vitamin D plus probiotic significantly reduced fasting plasma glucose (− 7.0 ± 9.9 vs. -0.2 ± 9.9 mg/dL, *P* = 0.01), insulin concentrations (− 2.7 ± 2.3 vs. + 0.4 ± 2.0 μIU/mL, *P* < 0.001), homeostasis model of assessment-estimated insulin resistance (− 0.8 ± 0.7 vs. + 0.1 ± 0.7, P < 0.001), triglycerides (− 7.8 ± 25.2 vs. + 10.1 ± 30.8 mg/dL, *P* = 0.01) and total cholesterol levels (− 4.9 ± 15.0 vs. + 5.9 ± 19.5 mg/dL, *P* = 0.04) and total−/HDL-cholesterol ratio (− 0.1 ± 0.6 vs. + 0.3 ± 0.8, P = 0.04).

**Conclusion:**

Probiotic and vitamin D for 12 weeks to chronic schizophrenia had beneficial effects on the general and total PANSS score, and metabolic profiles.

**Trial Registration:**

This study was retrospectively registered in the Iranian website (www.irct.ir) for clinical trials registration (http://www.irct.ir: IRCT2017072333551N2). 07-31-2017 2

## Background

Over 50 million people around the world suffer from schizophrenia [1]. It is the most disabling and costly chronic condition because treatment-resistant symptoms are very common [2]. Schizophrenia is a highly destructive illness characterized by recurrent relapses, cognitive decline, emotional and functional disability [3]. This disease consists of positive (hallucinations, delusions) and negative (emotional blunting, apathy) symptoms, and cognitive impairment, the two latter ones very resistant to antipsychotic medication [4]. With present alterations emerge to only show a modest benefit or do more disadvantage than good [5, 6], due to their side impacts and tolerance, there are calls to reconsider the impact that nutrition could have on mental disorders [7, 8]. In addition, metabolic disturbances including insulin resistance and dyslipidemia are related to cognitive impairment in schizophrenia which could help to functional decline found in these subjects [9].

It has been shown that crosstalk between nutrition and mental health is associated with neurotransmitter and hormonal pathways in the gut which regulate the function of brain [7]. Low levels Vitamin D is highly prevalent in schizophrenic patients [10, 11] and vitamin D supplementation might improve clinical and metabolic profiles in neuropsychiatric disorders, though available results are controversial. In a study, McGrath and colleagues [12] revealed that the supplementation of vitamin D could reduce the risk of schizophrenia at a dosage of at least 2000 IU/day in males in the first year of life. In a study conducted by Humble et al. [13], 85% of the 117 psychiatric subjects had suboptimal vitamin D status; interestingly the lowest level was for schizophrenic patients. The patients receiving vitamin D supplements achieved considerable improvement in their psychosis and depression symptoms [13]. However, vitamin D supplementation at a dosage of 1000 IU/day showed no changes in psychiatric symptoms [14]. Moreover, neuropsychiatric disorders, including schizophrenia and depression might be modulated by change in the exogenous probiotics and microbial substrates [15]. Probiotic supplementation for 14 weeks prevented common somatic symptoms of disease among outpatients with schizophrenia, yet did not affect the PANSS [16]. The single nutrient treatments including probiotic or vitamin D in the neuropsychiatric diseases show a modest sign enhancement or null findings [14, 16]. To date, there is a growing appeal to employ the combination platforms (e.g., vitamin D plus probiotics), particularly in subjects with vitamin D deficiency that could ameliorate symptoms in a number of mental psychiatric illnesses. The basis of this approach relies on probiotics effect on increasing vitamin D levels [17, 18].

### Aims of the study

Given the anti-inflammatory, antioxidant and immune-modulatory impacts of vitamin D and probiotics, we hypothesized that their combination might be beneficial in chronic schizophrenia. Therefore, conducted to evaluate the impacts of vitamin D and probiotic on clinical symptoms, biomarkers of oxidative stress and cardiometabolic risk in chronic schizophrenia.

## Methods

### Study design and population

This randomized, double-blind, placebo-controlled trial was registered by the Iranian registry of clinical trials (http://www.irct.ir: # IRCT2017072333551N2). It was conducted at a psychiatry clinic in Kashan, Iran from July to October 2017. Any participant who diagnosed with schizophrenia using DSM-IV-TR criteria with disease duration of at least two years, had PANSS score of 55 or greater, treated with chlorpromazine (300–1000 mg/day, except clozapine) and agents anticholinergic (Trihexyphenidyl, 4–8 mg/day) during the last 6 months and aged 25–65 years old were included in the study. Any subject with mental retardation (Intelligent Quotient of< 70), substance or alcohol addiction (except caffeine or nicotine) within the last 6 months of screening, a score of≥14 on a 17-item Hamilton Depression Rating Scale or a score of ≥4 on PANSS (depression item), anyone under treatment with lithium, carbamazepine, sodium valproic acid, with existing chronic and acute medical illness, with lactation or pregnancy, the use of antidepressants including MAO, TCA, SSRI in the last 6 months were excluded from this trials. Study protocol followed the principals of the Declaration of Helsinki and was approved by the ethics committee at Kashan University of Medical Sciences (KAUMS) (No. IR.KAUMS.REC.1396.10). Informed consent was signed by all participants prior to the intervention.

### Clinical trial procedures

Computer-generated random numbers were used by an instructed staff to randomize study participants at the psychiatry clinic. While randomizing, age (< 65 vs. ≥65 y), gender (male vs. female), BMI (< 25 vs. ≥25 kg/m^2^) and type and dosage of psychiatric medications were taken into consideration. Then, participants were equally allocated into either taking 50,000 IU of vitamin D3 every 2 weeks plus 8 × 10^9^ CFU/day of probiotic supplements containing *Lactobacillus acidophilus, Bifidobacterium bifidum, Lactobacillus reuteri,* and *Lactobacillus fermentum* (each 2 × 10^9^) for 12 weeks (treatment group, *n* = 30) or placebo which were capsules in the similar shape and packaging as vitamin D and probiotic (placebo group, n = 30). Participants were instructed not taking other vitamin D and probiotic supplements, while maintaining their regular diet and physical activity during the trial. Placebos, vitamin D, and probiotic were produced by Zahravi Pharmaceutical Company (Tabriz, Iran), LactoCare®, Zisttakhmir Company (Tehran, Iran) and Barij Essence Pharmaceutical Company (Kashan, Iran), respectively. Study compliance was determined by counting the remaining tablets in the returned containers as well as measuring serum 25-OH-vitamin D concentration. All participants were asked to record their food intake for 3 days (one and two weekdays) at week 1, 5, 9 and 12 of the trial. These recorded data were used to calculate participants’ nutrient intake for the Iranian food pattern. Physical activity was assessed through participants’ physical activity record at the first day of each month [19].

### Assessment

#### Biometric measures

The Height and weight of subjects were obtained without shoes and in light clothing by a trained staff in the psychiatry clinic at baseline and the end of the intervention. Body Mass Index (BMI) was reached as weight in kg divided by height in square meter.

#### Clinical measures

Severity of psychiatric symptoms was assessed using PANSS [20] and different domains of cognitive function were evaluated through BPRS scores [21]. These tests are routinely used for the assessment of cognition in schizophrenia in psychiatric settings worldwide. The PANSS is a 30-items rating scale composing of validated subscales to assess negative (7 items), positive (7 items), and general psychopathological (16 items) signs of schizophrenia. These 3 subscales are summed up in the PANSS total score [20]. BPRS was through using chart review and semi-structured clinical interview for DSM-IV-TR [21].

#### Biochemical measures

Fasting blood (10 mL) were collected from study participants at the beginning and after the 12-week intervention at Kashan reference laboratory (Kashan, Iran). Serum 25-hydroxyvitamin D concentrations were measured using an ELISA kit (IDS, Boldon, UK) with inter- and intra-assay coefficient of variations (CVs) below 7%. Total antioxidant capacity (TAC) concentrations were measured using the method of ferric reduction antioxidant power developed by Benzie and Strain [22]. Total glutathione (GSH) and malondialdehyde (MDA) concentrations also were measured using Beutler method [23] and thiobarbituric acid reactive substances spectrophotometric test, respectively [24]. CVs for plasma TAC, GSH and MDA were less than 5%. Serum high-sensitivity C-reactive protein (hs-CRP) levels were determined by commercial ELISA kit (LDN, Nordhorn, Germany) with inter- and intra-assay CVs below 7%. Nitric oxide (NO) levels were measured using Griess method [25]. Serum insulin concentrations were assessed through applying an ELISA kit (DiaMetra, Milano, Italy) with inter- and intra-assay CVs below 5%. The homeostasis model of assessment-insulin resistance (HOMA-IR) and the quantitative insulin sensitivity check index (QUICKI) were calculated using standard formula [26]. Enzymatic kits (Pars Azmun, Tehran, Iran) with intra-assay CVs of less than 5% were applied to measure fasting plasma glucose (FPG) and lipid profiles.

#### Statistical analyses

Suggested formula for clinical trial sample size calculation was used to calculate sample size in this study. Considering type one (α) and type two errors (β) of 0.05, and 0.20, of the power of study was 80%. According to Sheikhmoonesi et al. [27], we used positive PANSS score as the primary outcome with 5.4 as the mean change (d) and 6.7 as the SD. Based on the sample size calculation, 25 subjects were required for each treatment group; allowing for 20% dropouts, the final sample size was considered to be 30 participants in each group.

The Kolmogrov-Smirnov test was employed to check the normal distribution of data. Intention-to-treat (ITT) analysis was applied for all randomly allocated cases. Independent samples *t*-test was performed to compare the differences in baseline characteristics and daily dietary macro- and micro-nutrient intakes between the two treatment groups. Pearson Chi-square test was conducted for the comparison of categorical variables. To assess the impacts of vitamin D and probiotic on clinical symptoms, biomarkers of inflammation and oxidative stress, lipid profiles, insulin metabolism and repeated measures analysis of variance (RM ANOVA) was used. To control confounding variables, such as baseline values of each biochemical variable, age and baseline BMI, we were done analysis of covariance (ANCOVA). Significance level was the *p*-value of less than 0.05. Statistical Package for Social Science version 18 (SPSS Inc., Chicago, Illinois, USA) was applied to run all statistical analyses in this study.

## Results

**Fig. 1:**
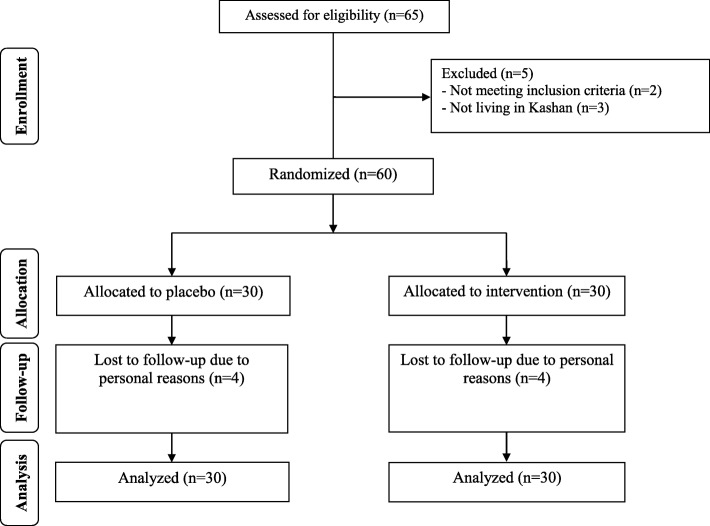
Summary of patient flow diagram

Four participants in the placebo groups and supplemented dropped out (Fig. 1). However, 60 participants were included in the final analysis using ITT methods. Generally, the compliance rate was high, because of more than 90% of capsules were consumed throughout the study in both groups and 25-hydroxyvitamin D levels significantly elevated in supplemented group.

There was no significant difference between the two groups in terms of height, age, weight, BMI and METs at baseline as well as mean changes in weight and BMI throughout the trial **(**Table 1**).**

**Table 1:** General characteristics of study participants at baseline study

	Placebo group (*n* = 30)	Vitamin D plus probiotic group (*n* = 30)	P^1^
Age (y)	43.2 ± 6.0	44.8 ± 8.3	0.41
Gender			
Female	2 (6.7)	2 (6.7)	> 0.99†
Male	28 (93.3)	28 (93.3)	
Height (m)	168.6 ± 7.5	170.6 ± 6.0	0.24
Weight at study baseline (kg)	69.5 ± 10.7	67.2 ± 9.0	0.38
Weight at end-of-trial (kg)	69.5 ± 10.5	67.5 ± 8.7	0.42
Body weight change (kg)	0.04 ± 0.7	0.2 ± 1.2	0.43
BMI at study baseline (kg/m^2^)	24.5 ± 3.7	23.1 ± 2.8	0.11
BMI at end-of-trial (kg/m^2^)	24.5 ± 3.7	23.2 ± 2.7	0.12
BMI change (kg/m^2^)	0.01 ± 0.2	0.1 ± 0.4	0.46

Data are means± SDs

^1^Obtained from independent samples *t*-test

^†^Obtained from Fisher’s exact test

Based on 3-days dietary records, we observed no significant difference in dietary energy, protein, carbohydrate, saturated fatty acids, fat, polyunsaturated fatty acids, monounsaturated fatty acids, total dietary fiber, cholesterol and micronutrients **(**Table 2**).**

**Table 2:** Mean dietary intakes of study participants at baseline, weeks 5, 9 and 12 of the study^1^

	Placebo group (*n* = 30)	Vitamin D plus probiotic group (*n* = 30)	P^2^
Energy (kcal/d)	2540 ± 184	2488 ± 147	0.23
Carbohydrates (g/d)	347.9 ± 42.1	337.9 ± 43.2	0.36
Protein (g/d)	89.5 ± 12.9	85.7 ± 11.2	0.23
Fat (g/d)	92.6 ± 13.4	92.2 ± 12.2	0.91
SFAs (g/d)	26.4 ± 5.6	26.3 ± 5.8	0.85
PUFAs (g/d)	30.0 ± 8.4	28.9 ± 6.9	0.61
MUFAs (g/d)	25.0 ± 5.6	24.4 ± 5.8	0.68
Cholesterol (mg/d)	188.6 ± 97.0	222.6 ± 119.7	0.18
TDF (g/d)	20.7 ± 5.2	18.9 ± 4.8	0.18
Magnesium (mg/d)	325.1 ± 88.3	295.8 ± 69.7	0.15
Zinc (mg/d)	11.1 ± 3.0	10.4 ± 2.8	0.34
Manganese (mg/d)	2.4 ± 0.8	2.3 ± 0.6	0.44
Calcium (mg/d)	1203.1 ± 161.9	1145.6 ± 231.3	0.27
Vitamin D (μg/d)	2.8 ± 0.7	2.6 ± 0.5	0.24

^1^Data are means± SDs

^2^Obtained from independent *t*-test

MUFAs, monounsaturated fatty acids; PUFAs, polyunsaturated fatty acids; SFAs, saturated fatty acids; TDF, total dietary fiber

After the 12-week intervention, vitamin D and probiotic led to a significant enhancement in 25-OH-vitamin D levels (+ 9.1 ± 4.1 vs. + 0.2 ± 0.4 ng/mL, *P* < 0.001), the general (− 3.1 ± 4.7 vs. + 0.3 ± 3.9, *P* = 0.004) and total PANSS score (− 7.4 ± 8.7 vs. -1.9 ± 7.5, *P* = 0.01) **(**Table 3**).** In addition, probiotic and vitamin D significantly increased plasma TAC (+ 51.1 ± 129.7 vs. -20.7 ± 53.3 mmol/L, *P* = 0.007), and decreased MDA (− 0.3 ± 0.9 vs. + 0.2 ± 0.4 μmol/L, P = 0.01) and serum hs-CRP levels (− 2.3 ± 3.0 vs. -0.3 ± 0.8 mg/L, *P* = 0.001). Moreover, taking vitamin D plus probiotic, significantly reduced FPG (− 7.0 ± 9.9 vs. -0.2 ± 9.9 mg/dL, *P* = 0.01), serum insulin concentrations (− 2.7 ± 2.3 vs. + 0.4 ± 2.0 μIU/mL, *P* < 0.001), HOMA-IR (− 0.8 ± 0.7 vs. + 0.1 ± 0.7, *P* < 0.001), triglycerides (− 7.8 ± 25.2 vs. + 10.1 ± 30.8 mg/dL, P = 0.01), total- (− 4.9 ± 15.0 vs. + 5.9 ± 19.5 mg/dL, *P* = 0.04), and total−/HDL-cholesterol ratio (− 0.1 ± 0.6 vs. + 0.3 ± 0.8, *P* = 0.04). Vitamin D and probiotic co-supplementation also significantly increased QUICKI (+ 0.02 ± 0.01 vs. + 0.0003 ± 0.01, P < 0.001). Taking vitamin D and probiotic had no significant effect on BPRS score and other metabolic profiles. Linear regression analysis revealed no association between serum 25-OH-vitamin D status and PANSS score at study baseline (β = 0.05, *P* = 0.16).

**Table 3:** Clinical symptoms, biomarkers of oxidative stress and cardio-metabolic risk at baseline and after the 12-week intervention in patients with schizophrenia

	Placebo group (*n* = 30)			Vitamin D plus probiotic group (*n* = 30)			P^1^
	Baseline	End-of-trial	Change	Baseline	End-of-trial	Change	
PANSS subscales							
Negative	29.1 ± 4.7	27.0 ± 3.8	−2.1 ± 3.7	27.8 ± 3.5	23.9 ± 5.0	−3.8 ± 4.1	0.08
Positive	19.4 ± 4.9	19.3 ± 5.3	−0.1 ± 2.8	22.8 ± 4.7	22.3 ± 4.8	−0.5 ± 3.8	0.64
General	38.9 ± 10.8	39.2 ± 9.5	0.3 ± 3.9	34.8 ± 6.4	31.7 ± 5.7	−3.1 ± 4.7	0.004
Total	87.5 ± 15.5	85.5 ± 14.1	−1.9 ± 7.5	85.4 ± 10.3	78.0 ± 11.3	−7.4 ± 8.7	0.01
BPRS	68.1 ± 9.7	68.5 ± 7.7	0.3 ± 4.9	58.5 ± 10.1	58.5 ± 10.2	0.0 ± 12.3	0.89
25-OH-vitamin D (ng/mL)	13.7 ± 3.2	13.9 ± 3.3	0.2 ± 0.5	15.0 ± 4.1	24.2 ± 5.3	9.1 ± 4.1	< 0.001
TAC (mmol/L)	1012.5 ± 208.6	991.7 ± 189.0	−20.7 ± 53.3	1014.1 ± 143.3	1065.3 ± 142.0	51.1 ± 129.7	0.007
GSH (μmol/L)	591.9 ± 103.2	627.3 ± 106.5	35.3 ± 93.8	681.3 ± 106.5	770.2 ± 129.3	88.9 ± 128.0	0.07
MDA (μmol/L)	2.5 ± 0.8	2.7 ± 0.7	0.2 ± 0.4	2.5 ± 0.9	2.2 ± 0.5	−0.3 ± 0.9	0.01
hs-CRP (mg/L)	4.6 ± 1.5	4.3 ± 1.7	−0.3 ± 0.8	4.3 ± 2.7	2.0 ± 1.5	−2.3 ± 3.0	0.001
NO (μmol/L)	37.8 ± 2.3	38.6 ± 2.7	0.9 ± 2.0	42.2 ± 4.0	42.6 ± 5.3	0.4 ± 3.0	0.43
FPG (mg/dL)	93.2 ± 9.4	93.0 ± 8.7	−0.2 ± 9.9	95.0 ± 10.5	88.0 ± 11.2	−7.0 ± 9.9	0.01
Insulin (μIU/mL)	12.1 ± 2.0	12.5 ± 2.7	0.4 ± 2.0	12.5 ± 3.7	9.8 ± 3.7	−2.7 ± 2.3	< 0.001
HOMA-IR	2.8 ± 0.5	2.9 ± 0.7	0.1 ± 0.7	2.9 ± 0.9	2.1 ± 0.8	−0.8 ± 0.7	< 0.001
QUICKI	0.32 ± 0.009	0.32 ± 0.01	0.0003 ± 0.01	0.32 ± 0.02	0.34 ± 0.02	0.02 ± 0.01	< 0.001
Triglycerides (mg/dL)	154.9 ± 62.2	165.0 ± 62.2	10.1 ± 30.8	142.6 ± 59.9	134.9 ± 49.7	−7.8 ± 25.2	0.01
VLDL-cholesterol (mg/dL)	31.0 ± 12.4	33.0 ± 12.4	2.0 ± 6.1	28.5 ± 11.9	27.0 ± 9.9	−1.5 ± 5.1	0.01
Total cholesterol (mg/dL)	172.6 ± 40.2	178.5 ± 35.8	5.9 ± 19.5	166.7 ± 35.9	161.8 ± 36.7	−4.9 ± 15.0	0.01
LDL-cholesterol (mg/dL)	101.2 ± 35.1	106.2 ± 32.5	5.0 ± 15.8	99.7 ± 28.2	96.6 ± 31.1	−3.0 ± 13.9	0.04
HDL-cholesterol (mg/dL)	40.4 ± 6.0	39.3 ± 6.3	−1.2 ± 6.3	38.5 ± 7.9	38.2 ± 9.2	−0.3 ± 5.7	0.59
Total−/HDL-cholesterol ratio	4.4 ± 1.3	4.7 ± 1.2	0.3 ± 0.8	4.4 ± 1.1	4.3 ± 1.2	−0.1 ± 0.6	0.04

All values are means± SDs

^1^*P* values represent the time × group interaction (computed by analysis of the one-way repeated measures ANOVA)

*BPRS* Brief Psychiatric Rating Scale; *FPG* Fasting plasma glucose; *GSH* Total glutathione; *HOMA-IR* Homeostasis model of assessment-estimated insulin resistance; *hs-CRP* High-sensitivity C-reactive protein; *MDA* Malondialdehyde; *NO* Nitric oxide; *PANSS* Positive and Negative Syndrome Scale; *QUICKI* Quantitative insulin sensitivity check index; *TAC* Total antioxidant capacity

There was observed a statically difference in the baseline levels of positive PANSS score (*P* = 0.009), BPRS (*P* < 0.001), GSH (*P* = 0.002) and plasma NO (P < 0.001). Hence, we adjusted the analysis for baseline values of biochemical parameters, age and baseline BMI. After controlling for these potential confounders, the difference in changes in total−/HDL-cholesterol ratio (*P* = 0.06) between the two groups became non-significant, while difference in changes in negative PANSS score (*P* = 0.03), BPRS (P = 0.03) and plasma GSH (*P* = 0.004) became statistically significant **(**Table 4**).** Other metabolic profiles did not change statically after this adjustment.

**Table 4:** Adjusted changes in metabolic variables in patients with schizophrenia

	Placebo group (*n* = 30)	Vitamin D plus probiotics group (*n* = 30)	P^1^
PANSS subscales			
Negative	−1.9 ± 0.6	−4.0 ± 0.6	0.03
Positive	−0.4 ± 0.6	−0.2 ± 0.6	0.82
General	0.7 ± 0.7	−3.5 ± 0.7	< 0.001
Total	−1.8 ± 1.4	−7.5 ± 1.4	0.007
BPRS	2.7 ± 1.6	−2.4 ± 1.6	0.03
25-OH-vitamin D (ng/mL)	0.2 ± 0.5	9.1 ± 0.5	< 0.001
TAC (mmol/L)	−22.3 ± 17.2	52.7 ± 17.2	0.004
GSH (μmol/L)	16.6 ± 20.4	107.7 ± 20.4	0.004
MDA (μmol/L)	0.2 ± 0.1	−0.3 ± 0.1	< 0.001
hs-CRP (mg/L)	−0.2 ± 0.3	−2.4 ± 0.3	< 0.001
NO (μmol/L)	0.8 ± 0.5	0.4 ± 0.5	0.61
FPG (mg/dL)	−0.7 ± 1.6	−6.5 ± 1.6	0.01
Insulin (μIU/mL)	0.5 ± 0.4	−2.7 ± 0.4	< 0.001
HOMA-IR	0.1 ± 0.1	−0.8 ± 0.1	< 0.001
QUICKI	0.0001 ± 0.003	0.01 ± 0.003	< 0.001
Triglycerides (mg/dL)	11.5 ± 4.9	−9.1 ± 4.9	0.005
VLDL-cholesterol (mg/dL)	2.3 ± 1.0	−1.8 ± 1.0	0.005
Total cholesterol (mg/dL)	6.6 ± 3.1	−5.6 ± 3.1	0.007
LDL-cholesterol (mg/dL)	5.1 ± 2.7	−3.1 ± 2.7	0.04
HDL-cholesterol (mg/dL)	−0.6 ± 1.1	−0.9 ± 1.1	0.87
Total−/HDL-cholesterol ratio	0.3 ± 0.1	−0.04 ± 0.1	0.06

All values are means± SEs. Values are adjusted for baseline values, age and BMI at baseline

^1^ Obtained from ANCOVA

*BPRS* Brief Psychiatric Rating Scale; *FPG* Fasting plasma glucose; *GSH* Total glutathione; *HOMA-IR* Homeostasis model of assessment-estimated insulin resistance; *hs-CRP* High-sensitivity C-reactive protein; *MDA* Malondialdehyde; *NO* Nitric oxide; *PANSS* Positive and Negative Syndrome Scale; *QUICKI* Quantitative insulin sensitivity check index; *TAC* Total antioxidant capacity

## Discussion

We found that probiotic and vitamin D for 12 weeks to patients with chronic schizophrenia had ameliorated effects on the general and total PANSS scores, as well as their metabolic profiles. This clinical trial reporting the effect of vitamin D and probiotic on clinical symptoms, biomarkers of oxidative stress and inflammation, lipid profiles and glycemic control in chronic schizophrenia. In current study, all patients were being hospitalized during the intervention; otherwise there was no difference in treatment protocol between outpatient and inpatient. The only difference might be the compliance rate which was closely monitored for inpatients. Moreover, to our best knowledge, the overall treatment protocol for schizophrenic patients is similar around globe. So, this study can be generalized to other countries as well. It should be taken into account that there was a significant difference in positive PANSS score and BPRS between placebo and vitamin D plus probiotic groups at study baseline. There are several reasons to explain this difference. In the current study, we did not randomize subjects based on their PANSS score and BPRS due to being diagnosed with chronic schizophrenia. Random allocation into two groups was done after stratification for age (< 65 vs. ≥65 y), gender (female vs. male) and pre-intervention BMI (< 25 vs. ≥25 kg/m^2^) by using computer random numbers. Therefore, the difference in positive PANSS score and BPRS existing in both groups occurred by chance. In the current study, there was no significant difference between two intervention groups in terms of weight and BMI after 12-week intervention. Further studies are required to examine the effects of probiotic and vitamin D on weight, BMI and metabolic outcomes in schizophrenia, considering longer duration, which may affect clinical symptoms.

### Effect on clinical signs

The current study showed that probiotic and vitamin D for 12 weeks to patients with chronic schizophrenia significantly ameliorated general and total PANSS scores, but did not impacts negative and positive PANSS scores, as well as BPRS score. Lack of significant effect of vitamin D and probiotic co-supplementation on BPRS and PANSS Positive subscales may be due to baseline values of measured PANSS subscales and BPRS, baseline status of 25-OH-vitamin D, different dosages and type of vitamin D and probiotic used. In order to improve BPRS and PANSS Positive subscales, individuals might need higher doses of vitamin D plus probiotic supplementation for a longer period of time to provide appropriate circulating levels for improving BPRS. Numerous evidences have reported that vitamin D deficiency remains a widespread difficulty in chronic schizophrenic [28–31]. Several risk factors for schizophrenia, including latitude, migration and season of birth, have been associated with vitamin D deficiency [28]. Furthermore, metabolic disorders such as diabetes, insulin resistance, obesity, cardiovascular disease and hyperlipidaemia, which are commonly show in chronic schizophrenic, might be related to low levels vitamin D. Serotonin, as one of the major neurotransmitters, has a crucial task in the function of brain [32]. Vitamin D, which controls more than 900 genes in the body, regulates brain serotonin synthesis through activating tryptophan hydroxylase 2 [33]. Low concentration of 25(OH)D in serum have been related to an elevated risk of attention deficit hyperactivity disease, schizophrenia, bipolar disorder, antisocial, and impulsive behavior [34, 35]. Available data suggest that 4000 IU/day of vitamin D may be able decrease the incidence of vitamin D deficiency and help decrease the risk of psychiatric diseases and improve brain function [36]. In another study, vitamin D supplementation to chronic schizophrenic patients, at a dosage of 14,000 IU biweekly, for 18 weeks was related a trend towards enhanced cognition, but did not influence psychosis, metabolic status or mood [37]. Probiotic microorganisms change the balance of gut microbiota and prevent abnormal responses to harmful food-derived antigens [38]. Schizophrenia is one of the diseases with increased levels of antibodies to food antigens [39]. Moreover, gastrointestinal issues such as constipation are very common in schizophrenic patients as the side effect of their medications and probiotics improve bowel function [40, 41]. Probiotics also modulate the inflammatory response and oxidative stress in the body [38, 42], which is one of the major pathologic grounds in schizophrenia [43]. These are the main reasons probiotics have been recommended in schizophrenic patients. There are animal studies showing that probiotic supplementation might change cognitive and behavioral abnormalities in sick animals [44, 45]; however, human studies are scarce. Current controversial evidence could be stated by different dosages of probiotics, various study designs, and vitamin D applied along with participants’ characteristics. The significant association between vitamin D deficiency and higher risk of schizophrenia has already been proved and the evidence regarding the modest impact of vitamin D supplementation on clinical symptoms of schizophrenia is there, however the magnitude was dependent upon an optimal background of other nutrients and helpful microbiota. Optimal vitamin D levels are requiring for nearly every cell; it is but one of the components required for proper functioning of all metabolic processed of the body. Multiple lines evidence proposing synergistic effect of combined vitamin D and administration of probiotic on clinical signs and metabolic diseases of subjects detected with schizophrenia. The basis of this approach relies on probiotics impact on elevating vitamin D concentrations [46]. Moreover, probiotics could have synergistic impacts with vitamin D, via enhancing vitamin D receptors expression [47].

### Effect on oxidative stress and inflammation

This study documented, taking probiotics and vitamin D for 12 weeks by patients with chronic schizophrenia significantly enhanced plasma TAC, and significantly reduced serum hs-CRP levels and plasma MDA, but did not affect TAC. Schizophrenia is associated with oxidative stress, inflammation and impaired antioxidant defense [46]. In healthy population and in those with different chronic diseases like diabetes, atherosclerosis and polyarthritis, the higher concentrations of 25(OH)D have been correlated with lower status of inflammatory biomarkers including interleukin 6, CRP, and tumor necrosis factor alpha [47, 48]. In a group of diabetic patients on hemodialysis, who were severely vitamin D deficient, inflammatory markers significantly decreased after 12 weeks of vitamin D supplementation [49]. In another study conducted by Anandabaskar et al. [50], high-dose vitamin D intake significantly improved reduced oxidative stress and vascular functions in type 2 diabetic patients. Similar impact has been shown in patients with MDD [51]. This effect might be influenced by different scales like disease condition, vitamin D dose, obesity and other nutrients status. Mousa et al. [52] did not find any lowering effect of vitamin D supplement on inflammation in obese adults supplemented with 4000 IU/day vitamin D for 16 weeks. The impacts of probiotics on oxidative stress and inflammation are controversial. *Lactobacillus casei* supplementation improved disease activity and inflammatory [53], though Mohammadi et al. [54] did not find any lowering effect of probiotics on inflammatory and oxidative stress markers. This discrepancy was the reason we speculate the synergistic effect of vitamin D with probiotics might end up with significant results.

### Effect on metabolic abnormalities

We found that vitamin D and probiotics for 12 weeks to chronic schizophrenia was related to a significant decrease in FPG, insulin, HOMA-IR, triglycerides, VLDL-, total-, LDL- and total−/HDL-cholesterol ratio, and a significant rise in QUICKI. Metabolic syndrome which is a cluster of different metabolic abnormalities including abdominal obesity, dyslipidemia, impaired fasting glucose, hypertension and or diabetes, is commonly prevalent in schizophrenic. According to National Cholesterol Education Program (NCEP) criteria, 41% of US in schizophrenia have metabolic syndrome [55]. Anti-inflammatory function of vitamin D might potentially decrease the incidence of insulin resistance and obesity in these patients [56, 57]. Among coronary artery disease, long-term vitamin D supplementation significantly reduced hyperglycemia and insulin resistance through attenuating oxidative stress and inflammation [58]. An eight-week pilot study was conducted in schizophrenic patients to assess the short-term impacts of vitamin D, 2000 IU/day, on their metabolic profile. The authors did not observe any significant changes in weight, glucose or lipid profiles. However, patients who had their serum 25(OH)D levels above 30 ng/mL at week 8 achieved a significantly greater reduction in total cholesterol levels [59]. Recent reported have depicted that gut microbiota play an important role in the pathogenesis of metabolic abnormalities and chronic diseases [60]. Probiotics can change gut flora, improve lipid profiles, and reduce blood glucose level and insulin resistance [60, 61]. Li et al. [62] showed in their meta-analysis that probiotic administration promoted glycemic control in diabetic patients. In another study, probiotic supplementation for 8 weeks improved lowered inflammatory and insulin sensitivity and oxidative stress. However, did not influence lipid profiles, fasting glucose, and TAC levels in patients with major depressive disorders [63, 64]. Nonetheless, Shimizu et al. [65] demonstrated the lowering effect of probiotics on lipid profiles as a potential approach for cardiovascular disease prevention. In order to address this discrepancy, the effect of probiotic and vitamin D should be taken into account.

This study is related to some limitations. In the current report, we did not characterize the microbiota and thus cannot establish whether probiotic administration over 12 weeks altered microbiota composition which leads to funding limitations. In addition, we could not evaluate an analysis of 16 s RNA of fecal matter. Therefore, analysis of 16 s RNA of fecal matter is suggested in future studies. Moreover, one cannot explain if the treatment impacts found in the present report was due to the impact of which component of the combined supplementation. Hence, future research is needed with single supplement in the present study in order to assess the ameliorate impacts of each supplement on clinical symptoms and metabolic profiles in chronic schizophrenic patients with longer duration of supplementation.

## Conclusions

Overall, probiotic and vitamin D for 12 weeks to chronic schizophrenia had impacts effects on the general and total PANSS scores, as well as their metabolic profiles. In addition, when we adjusted the analyses for baseline values, baseline BMI and age, the difference in changes of negative PANSS score and BPRS became statistically significant.

## Abbreviations

BPRS: Brief Psychiatric Rating Scale
FPG: Fasting plasma glucose
GSH: Total glutathione
HOMA-IR: Homeostasis model of assessment-estimated
hs-CRP: Insulin resistance, high-sensitivity C-reactive protein
MDA: Malondialdehyde
NO: Nitric oxide
PANSS: Positive and Negative Syndrome Scale
QUICKI: Quantitative insulin sensitivity check index
TAC: Total antioxidant capacity

## References

[1] Mitra S, Natarajan R, Ziedonis D, Fan X (2017). Antioxidant and anti-inflammatory nutrient status, supplementation, and mechanisms in patients with schizophrenia. Prog Neuro-Psychopharmacol Biol Psychiatry.

[2] Commission S (2012). The abandoned illness: a report from the schizophrenia commission (rethink mental illness, London).

[3] Chowdari KV, Bamne MN, Nimgaonkar VL (2011). Genetic association studies of antioxidant pathway genes and schizophrenia. Antioxid Redox Signal.

[4] Rabinowitz J, Levine SZ, Garibaldi G, Bugarski-Kirola D, Berardo CG, Kapur S (2012). Negative symptoms have greater impact on functioning than positive symptoms in schizophrenia: analysis of CATIE data. Schizophr Res.

[5] Moncrieff J (2007). Are antidepressants as effective as claimed? No, they are not effective at all. Can J Psychiatr.

[6] Martin A, Young C, Leckman JF, Mukonoweshuro C, Rosenheck R, Leslie D (2004). Age effects on antidepressant-induced manic conversion. Arch Pediatr Adolesc Med.

[7] Sarris J, Logan AC, Akbaraly TN, Amminger GP, Balanza-Martinez V, Freeman MP (2015). Nutritional medicine as mainstream in psychiatry. Lancet Psychiatry.

[8] Rucklidge JJ, Kaplan BJ, Mulder RT (2015). What if nutrients could treat mental illness?. Aust N Z J Psychiatry.

[9] Bora E, Akdede BB, Alptekin K (2017). The relationship between cognitive impairment in schizophrenia and metabolic syndrome: a systematic review and meta-analysis. Psychol Med.

[10] Valipour G, Saneei P, Esmaillzadeh A (2014). Serum vitamin D levels in relation to schizophrenia: a systematic review and meta-analysis of observational studies. J Clin Endocrinol Metab.

[11] Boerman R, Cohen D, Schulte PF, Nugter A (2016). Prevalence of vitamin D deficiency in adult outpatients with bipolar disorder or schizophrenia. J Clin Psychopharmacol.

[12] McGrath J, Saari K, Hakko H, Jokelainen J, Jones P, Jarvelin MR (2004). Vitamin D supplementation during the first year of life and risk of schizophrenia: a Finnish birth cohort study. Schizophr Res.

[13] Humble MB, Gustafsson S, Bejerot S (2010). Low serum levels of 25-hydroxyvitamin D (25-OHD) among psychiatric out-patients in Sweden: relations with season, age, ethnic origin and psychiatric diagnosis. J Steroid Biochem Mol Biol.

[14] Dealberto MJ (2013). Clinical symptoms of psychotic episodes and 25-hydroxy vitamin D serum levels in black first-generation immigrants. Acta Psychiatr Scand.

[15] Kim YK, Shin C. The microbiota-gut-brain axis in neuropsychiatric disorders: pathophysiological mechanisms and novel treatments. Curr Neuropharmacol. 2017. 10.2174/1570159X15666170915141036 [Epub ahead of print]. 10.2174/1570159X15666170915141036 PMC5997867 28925886

[16] Dickerson FB, Stallings C, Origoni A, Katsafanas E, Savage CL, Schweinfurth LA, et al. Effect of probiotic supplementation on schizophrenia symptoms and association with gastrointestinal functioning: a randomized, placebo-controlled trial. Prim Care Companion CNS Disord. 2014;16. 10.4088/PCC.13m01579 PMC4048142 24940526

[17] Jones ML, Martoni CJ, Prakash S (2013). Oral supplementation with probiotic L. reuteri NCIMB 30242 increases mean circulating 25-hydroxyvitamin D: a post hoc analysis of a randomized controlled trial. J Clin Endocrinol Metab.

[18] Shang M, Sun J (2017). Vitamin D/VDR, probiotics, and gastrointestinal diseases. Curr Med Chem.

[19] Ainsworth BE, Haskell WL, Whitt MC, Irwin ML, Swartz AM, Strath SJ (2000). Compendium of physical activities: an update of activity codes and MET intensities. Med Sci Sports Exerc.

[20] Risch SC, Horner MD, McGurk SR, Palecko S, Markowitz JS, Nahas Z (2007). Double-blind donepezil-placebo crossover augmentation study of atypical antipsychotics in chronic, stable schizophrenia: a pilot study. Schizophr Res.

[21] Lachar D, Bailley SE, Rhoades HM, Varner RV (1999). Use of BPRS-A percent change scores to identify significant clinical improvement: accuracy of treatment response classification in acute psychiatric inpatients. Psychiatry Res.

[22] Benzie IF, Strain JJ (1996). The ferric reducing ability of plasma (FRAP) as a measure of "antioxidant power": the FRAP assay. Anal Biochem.

[23] Beutler E, Gelbart T (1985). Plasma glutathione in health and in patients with malignant disease. J Lab Clin Med.

[24] Janero DR (1990). Malondialdehyde and thiobarbituric acid-reactivity as diagnostic indices of lipid peroxidation and peroxidative tissue injury. Free Radic Biol Med.

[25] Tatsch E, Bochi GV, Pereira Rda S, Kober H, Agertt VA, de Campos MM (2011). A simple and inexpensive automated technique for measurement of serum nitrite/nitrate. Clin Biochem.

[26] Pisprasert V, Ingram KH, Lopez-Davila MF, Munoz AJ, Garvey WT (2013). Limitations in the use of indices using glucose and insulin levels to predict insulin sensitivity: impact of race and gender and superiority of the indices derived from oral glucose tolerance test in African Americans. Diabetes Care.

[27] Sheikhmoonesi F, Zarghami M, Mamashli S, Yazdani Charati J, Hamzehpour R, Fattahi S (2016). Effectiveness of vitamin D supplement therapy in chronic stable schizophrenic male patients: a randomized controlled trial. Iran J Pharm Res.

[28] Chiang M, Natarajan R, Fan X (2016). Vitamin D in schizophrenia: a clinical review. Evid Based Ment Health.

[29] DeLuca GC, Kimball SM, Kolasinski J, Ramagopalan SV, Ebers GC (2013). Review: the role of vitamin D in nervous system health and disease. Neuropathol Appl Neurobiol.

[30] Samoes B, Silveira C (2017). The role of vitamin D in the pathophysiology of schizophrenia. Neuropsychiatry..

[31] Valipour G, Saneei P, Esmaillzadeh A (2014). Serum vitamin D levels in relation to schizophrenia: a systematic review and meta-analysis of observational studies. J Clin Endocrinol Metab.

[32] Lesch KP, Araragi N, Waider J, van den Hove D, Gutknecht L (2012). Targeting brain serotonin synthesis: insights into neurodevelopmental disorders with long-term outcomes related to negative emotionality, aggression and antisocial behaviour. Philos Trans R Soc Lond Ser B Biol Sci.

[33] Wang TT, Tavera-Mendoza LE, Laperriere D, Libby E, MacLeod NB, Nagai Y (2005). Large-scale in silico and microarray-based identification of direct 1,25-dihydroxyvitamin D3 target genes. Mol Endocrinol.

[34] Kinney DK, Teixeira P, Hsu D, Napoleon SC, Crowley DJ, Miller A (2009). Relation of schizophrenia prevalence to latitude, climate, fish consumption, infant mortality, and skin color: a role for prenatal vitamin d deficiency and infections?. Schizophr Bull.

[35] Tolppanen AM, Sayers A, Fraser WD, Lewis G, Zammit S, Lawlor DA (2012). The association of 25-hydroxyvitamin D3 and D2 with behavioural problems in childhood. PLoS One.

[36] Patrick RP, Ames BN (2014). Vitamin D hormone regulates serotonin synthesis. Part 1: relevance for autism. FASEB J.

[37] Krivoy A, Onn R, Vilner Y, Hochman E, Weizman S, Paz A (2017). Vitamin D supplementation in chronic schizophrenia patients treated with clozapine: a randomized, double-blind, placebo-controlled clinical trial. EBioMedicine..

[38] Dinan TG, Stanton C, Cryan JF (2013). Psychobiotics: a novel class of psychotropic. Biol Psychiatry.

[39] Dickerson F, Stallings C, Origoni A, Vaughan C, Khushalani S, Leister F (2010). Markers of gluten sensitivity and celiac disease in recent-onset psychosis and multi-episode schizophrenia. Biol Psychiatry.

[40] De Hert M, Dockx L, Bernagie C, Peuskens B, Sweers K, Leucht S (2011). Prevalence and severity of antipsychotic related constipation in patients with schizophrenia: a retrospective descriptive study. BMC Gastroenterol.

[41] Tomasik J, Yolken RH, Bahn S, Dickerson FB (2015). Immunomodulatory effects of probiotic supplementation in schizophrenia patients: a randomized. Biomark Insights.

[42] Bested AC, Logan AC, Selhub EM (2013). Intestinal microbiota, probiotics and mental health: from Metchnikoff to modern advances: part I - autointoxication revisited. Gut Pathog.

[43] Suvisaari J, Mantere O (2013). Inflammation theories in psychotic disorders: a critical review. Infect Disord Drug Targets.

[44] Diaz Heijtz R, Wang S, Anuar F, Qian Y, Bjorkholm B, Samuelsson A (2011). Normal gut microbiota modulates brain development and behavior. Proc Natl Acad Sci U S A.

[45] O'Mahony SM, Marchesi JR, Scully P, Codling C, Ceolho AM, Quigley EM (2009). Early life stress alters behavior, immunity, and microbiota in rats: implications for irritable bowel syndrome and psychiatric illnesses. Biol Psychiatry.

[46] Flatow J, Buckley P, Miller BJ (2013). Meta-analysis of oxidative stress in schizophrenia. Biol Psychiatry.

[47] Zittermann A (2003). Vitamin D in preventive medicine: are we ignoring the evidence?. Br J Nutr.

[48] Laird E, McNulty H, Ward M, Hoey L, McSorley E, Wallace JM (2014). Vitamin D deficiency is associated with inflammation in older Irish adults. J Clin Endocrinol Metab.

[49] Tamadon MR, Soleimani A, Keneshlou F, Mojarrad MZ, Bahmani F, Naseri A, et al. Clinical trial on the effects of vitamin D supplementation on metabolic profiles in diabetic hemodialysis. Horm Metab Res. 2017. 10.1055/s-0043-119221 [Epub ahead of print]. 10.1055/s-0043-119221 28958110

[50] Anandabaskar N, Selvarajan S, Dkhar SA, Kamalanathan SK, Tamilarasu K, Bobby Z (2017). Effect of vitamin D supplementation on vascular functions and oxidative stress in type 2 diabetic patients with vitamin D deficiency. Indian J Endocrinol Metab.

[51] Sepehrmanesh Z, Kolahdooz F, Abedi F, Mazroii N, Assarian A, Asemi Z (2016). Vitamin D supplementation affects the beck depression inventory, insulin resistance, and biomarkers of oxidative stress in patients with major depressive disorder: a randomized, controlled clinical trial. J Nutr.

[52] Mousa A, Naderpoor N, Johnson J, Sourris K, de Courten MPJ, Wilson K (2017). Effect of vitamin D supplementation on inflammation and nuclear factor kappa-B activity in overweight/obese adults: a randomized placebo-controlled trial. Sci Rep.

[53] Vaghef-Mehrabany E, Alipour B, Homayouni-Rad A, Sharif SK, Asghari-Jafarabadi M, Zavvari S (2014). Probiotic supplementation improves inflammatory status in patients with rheumatoid arthritis. Nutrition..

[54] Mohammadi AA, Jazayeri S, Khosravi-Darani K, Solati Z, Mohammadpour N, Asemi Z (2016). The effects of probiotics on mental health and hypothalamic-pituitary-adrenal axis: a randomized, double-blind, placebo-controlled trial in petrochemical workers. Nutr Neurosci.

[55] Meyer JM, Pandina G, Bossie CA, Turkoz I, Greenspan A (2005). Effects of switching from olanzapine to risperidone on the prevalence of the metabolic syndrome in overweight or obese patients with schizophrenia or schizoaffective disorder: analysis of a multicenter, rater-blinded, open-label study. Clin Ther.

[56] Arnson Y, Itzhaky D, Mosseri M, Barak V, Tzur B, Agmon-Levin N (2013). Vitamin D inflammatory cytokines and coronary events: a comprehensive review. Clin Rev Allergy Immunol.

[57] Zhang Y, Leung DY, Richers BN, Liu Y, Remigio LK, Riches DW (2012). Vitamin D inhibits monocyte/macrophage proinflammatory cytokine production by targeting MAPK phosphatase-1. J Immunol.

[58] Farrokhian A, Raygan F, Bahmani F, Talari HR, Esfandiari R, Esmaillzadeh A (2017). Long-term vitamin d supplementation affects metabolic status in vitamin D-deficient type 2 diabetic patients with coronary artery disease. J Nutr.

[59] Thakurathi N, Stock S, Oppenheim CE, Borba CP, Vincenzi B, Seidman LJ (2013). Open-label pilot study on vitamin D(3) supplementation for antipsychotic-associated metabolic anomalies. Int Clin Psychopharmacol.

[60] Li Z, Yang S, Lin H, Huang J, Watkins PA, Moser AB (2003). Probiotics and antibodies to TNF inhibit inflammatory activity and improve nonalcoholic fatty liver disease. Hepatology..

[61] Guo Z, Liu XM, Zhang QX, Shen Z, Tian FW, Zhang H (2011). Influence of consumption of probiotics on the plasma lipid profile: a meta-analysis of randomised controlled trials. Nutr Metab Cardiovasc Dis.

[62] Li C, Li X, Han H, Cui H, Peng M, Wang G, et al. Effect of probiotics on metabolic profiles in type 2 diabetes mellitus: A meta-analysis of randomized, controlled trials. Medicine (Baltimore). 2016;95:e4088. 10.1097/MD.0000000000004088 PMC4937966 27368052

[63] Akkasheh G, Kashani-Poor Z, Tajabadi-Ebrahimi M, Jafari P, Akbari H, Taghizadeh M (2016). Clinical and metabolic response to probiotic administration in patients with major depressive disorder: a randomized, double-blind, placebo-controlled trial. Nutrition..

[64] Mansournia MA, Ostadmohammadi V, Doosti-Irani A, Ghayour-Mobarhan M, Ferns G, Akbari H (2018). The effects of vitamin D supplementation on biomarkers of inflammation and oxidative stress in diabetic patients: a systematic review and meta-analysis of randomized controlled trials. Horm Metab Res.

[65] Shimizu M, Hashiguchi M, Shiga T, Tamura HO, Mochizuki M (2015). Meta-analysis: effects of probiotic supplementation on lipid profiles in normal to mildly hypercholesterolemic individuals. PLoS One.

